# Interarch comparison of intraoral pH and temperature: a pilot study

**DOI:** 10.1038/bdjopen.2016.8

**Published:** 2016-11-25

**Authors:** Jung Eun Choi, Karl M Lyons, Mitten CB McLean, Neil J Waddell

**Affiliations:** 1 Sir John Walsh Research Institute, Faculty of Dentistry, University of Otago, Dunedin, New Zealand

## Abstract

**Purpose of study:**

The severity of tooth wear is known to have an association with intraoral pH and temperature depending on the site.

**Objective:**

To compare the intraoral pH and temperature between the maxillary and mandibular arch.

**Methods:**

Fourteen participants (mean age=25.8 years) wore a custom-made intraoral appliance fitted with a pH probe and thermocouple for 24 h while carrying out normal activities including sleep. All participants wore a maxillary appliance; four participants repeated the process and wore the mandibular appliance. Measurements were taken from the palatal aspect of the upper central incisors and lingual aspect of the lower canines. Both qualitative and quantitative statistical analyses were conducted.

**Results:**

The mean intraoral pH from the maxilla was 7.32 (±0.52) and 7.07 (±0.26) for the mandible. During daytime, there was no statistical significance difference between the two arches (*P*=0.12). During sleep, there was a significant difference (*P*<0.001) between the mean pH of the maxilla, 7.0 (±0.46), and mandible, 6.46 (±0.31). The fluctuation patterns of pH and temperature from both arches were similar, but the maxilla showed more variations. The mean temperature from the mandible was slightly higher (36.18 °C (±0.96)) than the maxilla, 33.12 °C (±5.51) during daytime; however, there was no statistically significance difference in temperature between the arches during daytime (awake) or sleep (*P*=0.27).

**Conclusion:**

The results showed that there is significant difference in mean intraoral pH between the maxilla and the mandible during sleep, but not during the day and this difference may be associated variations in tooth wear between the arches.

## Introduction

Dental erosion is a chemical tooth wear caused by a decrease in intraoral (salivary) pH. It is a significant issue in dentistry since it affects 4–82% of the adult population.^1,2^ Tooth erosion is a multifactorial condition and has a complex aetiology in which the acids involved in the chemical dissolution may be of intrinsic and extrinsic origins.^3^ Previous studies have revealed that externally consumed acids from low pH beverages, such as fruit-based drinks and sports drinks, play a significant role in the erosion of enamel and dentine. The intrinsic acid caused by gastroesophageal reflux disease and self-provoked gastric regurgitation has also been found to have a strong relationship with dental erosion.^2^ Depending on the aetiology, erosion is found to develop in different sites around patients’ teeth^4,5^ and the site specificity of the condition is a particular concern for clinicians and dental researchers.

The distribution of erosion is influenced by factors, which increase or reduce the chance of the contact between the source of acid and tooth surfaces.^4^ It has been found that individuals with a habit of swishing a drink around the teeth before swallowing tend to have erosion on the labial/buccal side of the teeth.^4,6,7^ In contrast, patients with eating disorders or gastroesophageal reflux disease are found to have dental erosion in the palatal aspect of the teeth.^8,9^ The main determining factor for the site-specificity of erosion is however the saliva. Saliva protects the teeth by diluting and neutralising or clearing buffering acids, and by forming a protective pellicle to reduce the demineralisation rate and to enhance remineralisation. The saliva secreted from different locations (the parotid, submandibular and sublingual) in the mouth varies in composition, and this has been found to influence dental erosion to various degrees.^7,10^ Dawes *et al.*^10,11^ stated that the reduced salivary flow rate retards oral sugar clearance, which adversely affects saliva buffering of the acid present in the mouth. Acid clearance by saliva is also affected by food consistency and the site of the mouth, with sites poorly bathed by saliva are more likely to show erosion compared with those areas protected by saliva. This may be one of the reasons why the facial surfaces of upper incisors are more susceptible to erosion when the opposite is true for the lingual surfaces of the lower teeth.^6^ The prevalence of dental erosion in those with salivary flow impairment further supports the importance of saliva as an oral defence against dental erosion.^12^

Previous studies that have investigated intraoral pH have limitations however. First, studies reporting the site-specificity and prevalence of erosion have used different indices and criteria.^1,13^ This makes it difficult to compare results and extrapolate the causal factors. Second, salivary variables (pH/buffering capacity and flow rate) are traditionally measured from saliva collected extraorally, which can be inaccurate since a more general pH is produced, that does not represent the real-time variation in the intraoral environment that affects the development of erosion.^5,14^

Our research group have developed and validated an intraoral appliance that enables the continuous and simultaneous measurement of pH and temperature up to 48 h.^15^ Previous studies have confirmed that there is a difference in intraoral pH and temperature during sleep compared with daytime values,^15,16^ however, these studies only took measurements from the palatal aspect of the upper central incisors. Investigating whether there is a difference and a variation in the pH between the arches when measured continuously would provide useful information that can be used as baseline data for investigating the site specificity of dental erosion. Although temperature does not have a direct relationship with erosion, it has been found to influence the reactivity and the outcome of chemical reactions occurring in the mouth.^15,17^ Furthermore, there is no study yet which has compared the intraoral temperature between two arches over a 24-h period.

The objective of this study was to measure and compare intraoral pH and temperature from two different sites (mandible and maxilla) to investigate whether there is an interarch difference between these two variables.

## Methods

### Participant recruitment

After ethical approval (University of Otago Human Ethics Committee H14/012) was obtained, 14 healthy participants agreed to participate in the study. Volunteers were recruited from the students and staff body at the University of Otago, Dunedin, New Zealand. Subjects were selected after completing a health status questionnaire that identified the following exclusion criteria; history of dental erosion, xerostomia, eating disorders, respiratory disorders, sleep disorders, allergy, intake of medication, mouth breathing, smoking, wearing of orthodontic appliances and restorations on the upper and lower teeth.

**Figure 1:**
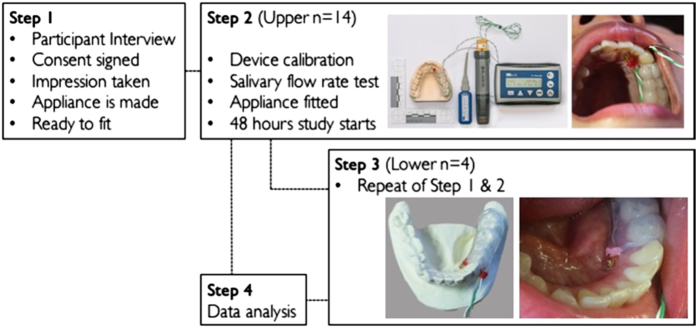
Study flow chart showing steps 1–3 of the recording process with images of intraoral appliance constructed to measure pH and temperature.

Participants (mean age=25.8 years) were asked to be present at the University of Otago Faculty of Dentistry dental clinic and alginate impressions of the upper teeth were taken. Four of the 14 participants agreed to wear the customised appliance for the lower arch, and for these participants an impression of the lower teeth was also taken. Custom-made appliances were made as described in a previous study.^15^ A vacuum-formed appliance covering the first quadrant of the maxilla was fitted with a pH measurement probe (ResTech, San Diego, CA, USA) and a thermocouple (K-Type, Lascar Electronics, Erie, PA, USA). The probe and the thermocouple were placed 3–5 mm behind the central incisors (Figure 1). For the lower device, the pH probe and thermocouples were placed 3–5 mm behind the lower lateral incisors. Before the appliances being fitted, participants’ salivary flow rate was measured using the 5 min spit technique.^18^ The appliances were calibrated and fitted intraorally and worn by the participants for 24 h while carrying out normal daily activities including sleep (Figure 1, step 2).

The appliances were taken off when eating and washing to avoid water damaging the data transmitters. A minimum of 1 week after the first set of experiments, participants were asked to wear a newly made and calibrated device again and repeat the experimental process (Figure 1, step 3). Participants were also asked to keep a detailed log of daily activities during the study participation days. Once completed, the results stored in a SD card or USB were retrieved and analysed via computer software (View Lite, ResTech).

### Data analysis

The recording taken when the intraoral appliance was not worn (e.g., meal times and shower) were tracked according to the daily logs provided by the participants and were subsequently deleted. The study data were then categorised into groups and measurement phases (awake/sleep) and the categorised data were summarised using descriptive statistics (mean, minimum and maximum). Estimation of the variance was investigated as the total s.d. as well as a coefficient of variation and an independent sample *t*-test was conducted. All statistical analyses were performed using SPSS version 22 for Windows (SPSS, Chicago, IL, USA) and *P*-values <0.05 were regarded as being statistically significant.

## Results

The results obtained from the current study are summarised in Table 1. The mean pH during daytime measured from maxilla was 7.32±0.52, whereas that of mandible was 7.07±0.26 (*P*=0.12. t=1.12). During sleep, there was a significant difference (*P*=0.01, t=2.82) between the mean pH of the maxilla, 7.0±0.46, and mandible, 6.46±0.31.

**Figure 2:**
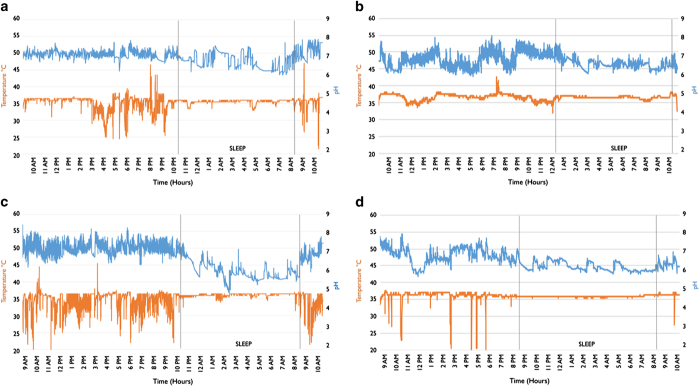
Typical pH and temperature graphs on two subjects P #2 (**a**, **b**) and P #5 (**c**, **d**) over a day period. Graph (**a**, **c**) are obtained from the upper arches of the participants, whereas graph (**b**, **d**) are obtained from the lower arches. Note the difference in pattern of variation of pH and temperature during daytime and sleep and between the two arches. For each graph: the *y* axis to the left indicates temperature; the *y* axis to the right indicates pH; the *x* axis indicates time. The upper line indicates pH (blue); the lower line indicates temperature (orange).

The fluctuation patterns of pH from maxillary and mandibular arches were found to be similar, however, the pH measured from the upper teeth had more variation throughout the day, including sleep (CoV during daytime 7.1%, sleep 6.57), compared with the lower arch (3.67%, 4.79%). These patterns are depicted in the graphs in Figure 2.

The mean temperature from the mandible was slightly higher (36.18 °C±0.96) than the maxilla, 33.12 °C (±5.51) during daytime. There was no statistical significance in temperature between the arches during daytime (*P*=0.16, *t*=1.44). During sleep, the temperature for the upper increased slightly, whereas that from lower decreased slightly although there was no statistical significance (*P*=0.27, *t*=1.1). A noticeable difference was found in the CoV of temperature for the upper; the CoV during daytime was 16.6/18.7%, and the CoV of the lower was 2.68 and 1.94%. The mean temperature measured from the upper arch showed 6–9 times higher variation compared with that measured from the mandible.

## Discussion

Numerous risks factors have been suggested to influence the severity of tooth wear and the relationship among these factors and tooth wear is typically complex. Saliva is considered the most important defence factor that protects the teeth from erosive tooth wear. Both quantity and quality of saliva are thought to be contributing factors in the tooth wear process.^1,2^ Dawes^11^ reviewed the possible reasons when saliva may have a limited capacity to protect the teeth against erosion and found that salivary characteristics including salivary acid clearance, buffering capacity, pH and thickness of salivary pellicle were all factors. Because of the importance of saliva, previous studies have used a wide range of methods to measure salivary variables, especially the salivary pH, that have ranged from lab-based experiments with collected saliva to in-clinic diagnostic tests to spot-measuring devices.^6,10^ However, the limitations of these methods were that they do not record the real-time variation in pH and there was a chance of interrupting the circadian rhythm depending on the time of measurement and collection of saliva.^15,16^

Salivary variables have also been found to influence the distribution and severity of erosion. Since the saliva secreted from different locations in the mouth varies in composition, dental erosion may be found to be present in various degrees accordingly. Previous studies have revealed that the palatal aspect of central incisors is the area found with the most erosion, whereas it is the opposite for lingual aspects of lower teeth, due to the lowered salivary pH, buffering capacity and acid clearance around the palatal aspects of upper teeth compared with the lingual aspects of lower teeth.^4,5,7^

One of the findings in this study was opposite to that which had been reported in previous studies. The observed difference in the pH between the maxilla and the mandible during the day was not significant. There was a significant difference found in the pH from the two arches during sleep, however, the pH from the mandible was found to be lower than the maxilla when it has previously been reported that the pH in the maxilla was more acidic.^5,14^ The difference in findings may have occurred because of the time of measurement in previous studies. Most studies have measured introral pH during the day, and only for a short periods but not during sleep.^1^ Yosipovitch *et al.*^14^ speculated that the hard palate should have an unstable pH compared with the lower, since it is the area where the thinnest saliva film is covering the tissue and mouth breathing induces evaporation, which may rapidly decrease the thickness of the salivary, leading to the rapid changes of pH around the site. In the current study, saliva pH was monitored continuously over 24 h and confirmed the speculation that pH measured from maxillary arch was unstable compared with the mandibular pH. Despite a similar range of pH in the maxilla and mandible during the day, there was a significant difference in CoV results; maxillary pH had a 7.1% variation during the day compared with the lower pH that had almost half the variation (3.67%). This trend was also observed while participants were sleep.

Intraoral temperature in upper arch was also found to have a significant coefficient of variation throughout the day compared with mandible. Although the intraoral temperature values were not significantly different, the temperature variation was 6–9 times higher in the maxilla than the mandible. This may be due to the direction of airflow during mouth breathing, eating and speaking during daytime compared with mainly breathing during sleep. The large s.d. and coefficient of variation indicates the fluctuation in intraoral temperature due to opening and closure of the mouth for various activities, such as breathing, eating and speaking. It has been found that intraoral temperature is lowered on inhalation, due to ‘evaporative effects’ during breathing, whereas the temperature is close to body temperature during exhalation, which explains the great fluctuation in intraoral temperature during the day.^16^ Mouth breathing is known to affect the pH and temperature in the mouth as a result of its evaporative effects and dehydration of saliva during breathing, and a previous study by Choi *et al.*^16^ has confirmed that mouth breathing causes a greater fluctuation of the pH and temperature than nasal breathing. The direction of the airflow when we mouth breathe follows the palatal contour of the upper incisors, whereas the lower teeth are protected by the lip and the tongue, and this may have contributed to the difference in the CoV of pH and temperature observed from maxilla and mandible.^16^ This means that the upper teeth may not be more susceptible to erosion just because of a decrease in pH, but also because of the unstable pH created in the oral environment by various other factors.

The current study was limited in several ways. The number of participants who wore the lower device was very small. Although a sample calculation conducted before the study revealed that having four participants was enough to show the significance difference between the arches with the power of 0.8, however, having more participants in the future would increase the power of the data and enable a stronger observation of variation patterns of pH and temperature. Another limitation was that only one site from each arch was chosen for measurements. Although the interarch variation of pH and temperature was observed over an extended period, having the measurement taken from one site from each arch may not be enough to represent the variation occurring in the maxilla and mandible. Therefore, future studies measuring intraoral pH and temperature from multiple sites will provide a better understanding of the real-time changes in the oral cavity. Further studies having participants to consume different drinks might also help our understanding of the real-time change of buffering capacity and salivary change in different sites in the oral cavity. Moreover, studies investigating interarch variation of pH and temperature in patient groups that show site specificity of erosion will also be valuable.

## Conclusion

Within the limitations of this study, the following conclusions were drawn: there is an interarch difference in intraoral pH and a noticeable difference in the pattern of variation of pH and temperature between maxilla and mandible when measured over a 24-h period. Also, the intraoral pH from the mandible decreases during sleep compared with maxilla.

## Figures and Tables

**Table 1:** Mean, s.d., maximum, minimum, coefficient of variation (s.d./mean×100) of intraoral pH and temperature measure from upper and lower arches of participants

	*pH*				*Temperature*			
	*Upper*		*Lower*		*Upper*		*Lower*	
	*Awake*	*Sleep*	*Awake*	*Sleep*	*Awake*	*Sleep*	*Awake*	*Sleep*
Mean (±s.d.)	7.32±0.52	7.00±0.46	7.07±0.26	6.46±0.31	33.12±5.51	33.37±6.25	36.18±0.97	36.03±0.70
Maximum	8.08	6.16	8.75	8.08	36.41	36.53	55	38
Minimum	6.8	7.8	4.92	5.66	13.29	10.85	9.5	24
CoV (%)	7.1	6.57	3.67	4.79	16.6	18.7	2.68	1.94
